# Decision making, central coherence and set-shifting: a comparison between Binge Eating Disorder, Anorexia Nervosa and Healthy Controls

**DOI:** 10.1186/s12888-015-0395-z

**Published:** 2015-01-24

**Authors:** Matteo Aloi, Marianna Rania, Mariarita Caroleo, Antonella Bruni, Antonella Palmieri, Maria Antonella Cauteruccio, Pasquale De Fazio, Cristina Segura-García

**Affiliations:** Chair of Psychiatry. Department of Health Sciences, University Magna Graecia of Catanzaro, Catanzaro, Italy; Department of Education of Calabria Region, Catanzaro, Italy

**Keywords:** Anorexia Nervosa, Binge Eating Disorder, Cognitive flexibility, Central coherence, Set-shifting, Decision making, Neuropsychology, Depression

## Abstract

**Background:**

Several studies have investigated the cognitive profile in patients with Anorexia Nervosa (AN) and Bulimia Nervosa (BN); on the contrary few studies have evaluated it in patients with Binge Eating Disorder (BED). The purpose of this study was to compare decision making, central coherence and set-shifting between BED and AN patients.

**Methods:**

A battery of neuropsychological tests including the Iowa Gambling Task (IGT), the Rey-Osterrieth Complex Figure Test (RCFT), the Wisconsin Card Sorting Test (WCST), the Trial Making Task (TMT) and the Hayling Sentence Completion Task (HSCT) were administered in a sample of 135 women (45 AN, 45 BED, 45 Healthy Controls [HC]). Furthermore, Beck Depression Inventory (BDI) was administered to evaluate depressive symptoms. Years of education, age, Body Mass Index (BMI) and depression severity were considered as covariates in statistical analyses.

**Results:**

BED and AN patients showed high rates of cognitive impairment compared to HC on the domains investigated; furthermore, the cognitive profile of BED patients was characterised by poorer decision making and cognitive flexibility compared to patients with AN. Cognitive performance was strongly associated with depressive symptoms.

**Conclusions:**

In the present sample, two different neurocognitive profiles emerged: a strong cognitive rigidity and a central coherence based on the details was predominant in patients with AN, while a lack of attention and difficulty in adapting to changes in a new situation seemed to better describe patients with BED. The knowledge of the different cognitive profiles of EDs patients may be important for the planning their psychotherapeutic intervention.

## Background

Cognitive flexibility (i.e., the mental ability to adjust thinking or attention in response to changing goals and/or environmental stimuli) has been widely studied in patients with Eating Disorders (EDs) [1,2]. Most of these studies were conducted with female patients with Anorexia Nervosa (AN) or Bulimia Nervosa (BN) demonstrating strong cognitive rigidity [3,4]. Inflexibility is a typical feature of AN [5] and is evident in ED related thoughts (e.g., categorising food as good or bad, thinking about calories, fat or sugar content, etc.), behaviours (e.g., calculating calories consumed and/or burned, continuous body shape and weight control, ritualised exercise pattern, etc.) and difficulties in finding alternative ways of dealing with a problem. Interestingly, extreme weight condition (EWC) groups like AN patients and obese subjects (OB) have shown similar dysfunctional executive profiles [6,7].

Binge Eating Disorder (BED) is an ED characterised by recurring episodes of over eating associated with lack of control during which patients eat large amounts of food [8]. In contrast to BN, recurrent inappropriate compensatory behaviours to counterbalance the consequences of bingeing are absent, for this reason patients with BED are frequently obese. Several studies have shown that BED patients have a higher lifetime prevalence of other psychiatric disorders such as personality [9], anxiety and mood disorders [10]. Few studies have assessed cognitive flexibility among patients with BED; in all cases BED patients were compared to OB [11-16].

A recent functional MRI study has shown neurocognitive impairment in BED, particularly in the reward circuitry of the brain [17]. More specifically, BED patients showed diminished recruitment of the ventral striatum and the inferior frontal gyrus during the anticipatory phase of reward processing and reduced activity in the medial prefrontal cortex during the outcome phase of reward processing. In three studies [12,15,16], obese individuals with BED showed impaired decision-making compared with people without BED [12], and BED overweight women performed more poorly on neuropsychological testing than people without BED [15,16]. However, in other studies [13,14] no significant differences emerged between people with and without BED on cognitive testing.

Studies have shown that people with cognitive impulsivity (i.e., the inability to weigh the consequences of immediate and future events and, consequently, inability to delay gratification) like BED patients, found themselves in trouble in certain situations because their decision-making pattern was influenced by immediate rewards [13,18]; furthermore attentional/executive deficit played an important role in the development and maintenance of obesity and EDs [19,20]. In fact, the possible comorbidity between BED and Attention Deficit Hyperactivity Disorder (ADHD) - characterised by symptoms of impulsivity, hyperactivity and inattention - has been highlighted [21,22].

Based on the above, the purpose of the present study was to assess and compare BED and AN patients on the three most widely studied domains of cognition (i.e., set-shifting, central coherence and decision-making) through neuropsychological testing. We hypothesised that, similarly to AN patients, BED patients might also exhibit a pathological but different pattern of cognition in the following way: BED patients would exhibit more errors in the decision-making task, more non-perseverative errors in the set-shifting domain and poorer central coherence than AN patients.

## Methods

### Patients and procedures

The sample for this study consisted of 135 female participants: 45 patients with AN Restrictive type, 45 patients diagnosed with BED and 45 healthy controls (HC). Patients were consecutively recruited at an Ambulatory for Treatment of EDs and were diagnosed by experienced clinicians according to DSM-IV-TR [8] diagnostic criteria, using the structured clinical interview for DSM IV Axis I disorders (SCID-I) [23].

The interviewers were clinicians who worked in the field of EDs who were trained in the administration of SCID and neuropsychological tests and used these tools in their daily clinical practice. An expert psychologist in this field supervised the interviewers during the data collection.

HC participants were recruited at the local University and the Socio-Psycho-Pedagogical High School of the city. Prior to assessment, they were all interviewed and asked about the lifetime presence of an ED or other axis I diagnosis (i.e. mood disorder, anxiety disorder, psychotic disorder, impulse-control disorder), history of being underweight [Body Mass Index (BMI) <17.5] and obesity (BMI ≥30) and were excluded if so. Patients and HC were all Caucasian.

Inclusion criteria in the clinical groups were: AN Restrictive type or BED diagnosis according to DSM-IV TR. All participants were subject to the following exclusion criteria: a) male gender, b) drug dependence, c) use of psycho-active medications, d) cognitive deficits as indicated by Mini Mental State Examination score <24 [24], e) history of chronic medical illness or neurological condition that might affect cognitive function, f) head trauma with loss of consciousness for more than 2 minutes, g) other severe medical comorbidity (e.g., epilepsy or diabetes), h) BMI < 14 for AN patients. Furthermore, following the indications of other researchers, individuals older than 45 years were excluded [13,14].

Participants were informed about the aim of the study, that participation was voluntary, that personal data would be kept confidential and that no extra credit would be given to students for their participation. All partakers, or their parents on behalf of those younger than eighteen, signed a written informed consent according to the Ethical Committee, before entering the study. The study, approved by the Ethical Committee of Azienda Ospedaliera Universitaria Mater Domini, was conducted from July 2013 to June 2014.

### Measures

#### Neuropsychiatric assessment

ED psychopathology was assessed by means of the Eating Disorder Inventory-2 (EDI-2) [25] and the Binge Eating Scale (BES) [26] and depression severity was evaluated with the Beck Depression Inventory (BDI) [27].

##### EDI-2

The EDI-2 is a self-report questionnaire that assesses the psychopathology of EDs using 91 items on a six-point Likert-type scale from 0 ‘never’ to 6 ‘always’ coded with a 3-point system where ‘sometimes’ , ‘rarely’ and ‘never’ were assigned zeros while ‘often’ , ‘usually’, and ‘always’ were assigned a score of 1, 2 and 3, respectively. Subscales measured by the test were: Drive for Thinness (DT), Bulimia (B), and Body Dissatisfaction (BD), Perfectionism (P), Interoceptive Awareness (IA), Maturity Fears (MF), Ineffectiveness (I), and Interpersonal Distrust (ID), Asceticism (A), Social insecurity (SI), and Impulse Regulation (IR). Cronbach’s alpha in the present study was 0.91.

##### BES

The BES is an easily administered test with adequate internal consistency and validity that has been widely used in research either to measure binge eating severity in the non-purge binge eating population or to determine whether potential research participants meet the inclusion criteria of binge eating. It is made up of 16 items describing the behavioural manifestations, feelings and cognitions associated with binge eating. Each item consisted of four statements that reflected a range of severity from which subjects chose the one that best described perceptions and feelings about their own eating behaviour. Total BES score <17 indicated unlikely BED, 17–27 score possible BED and values >27 probable BED. BES was only administered to BED patients in order to further corroborate the diagnosis. Cronbach’s alpha in this study was 0.89.

##### BDI

The BDI self-report questionnaire is widely used to assess the severity of depressive symptoms and consisted of 21 items. The first thirteen items assessed the cognitive-affective sphere and the remaining eight converged into the performance-somatic symptoms scale. The clinical cut-off has been set at 16; scores between 0–9, 10–16, 17–29 and ≥30 indicated minimum, mild, moderate and severe depression respectively. Cronbach’s alpha in this study was 0.91.

#### Anthropometric measures

Participants, wearing light indoor clothing and no shoes, were measured using a portable stadiometer (Seca 220, GmbH & Co., Hamburg, Germany) and a balance scale (Seca 761, GmbH & Co., Hamburg, Germany); their standing height to the nearest 0.1 cm and body weight to the nearest 0.1 kg were taken the morning of the assessment. Then the individual’s BMI (= kg^.^m^−2^) was calculated.

#### Neuropsychological assessment

All participants were assessed with the following neuropsychological tests: a) Iowa Gambling Task (IGT) [28]; b) Rey-Osterrieth Complex Figure Test (RCFT) [29]; c) Trial Making Task (TMT) [30]; d) Wisconsin Card Sorting Test (WCST) [31]; e) Hayling Sentence Completion Test (HSCT) [32].

##### IGT

The computerised version of the original IGT was used to assess decision making. The subject was given a virtual amount of money to play with; the task required selecting one hundred cards from four decks. By selecting one card over another, the participant can either gain or gain-and-lose virtual money. Decks A and B were disadvantageous in the long run because the total gain was lower than the total loss, whereas decks C and D were advantageous because although the gains were lower, the penalties were also lower. The goal of the task was to make the most profit. Decision-making ability was determined by examining IGT performance over time; this was done by dividing the 100 card choices in five blocks of 20 trials. Performance was measured by calculating a ‘net score’ for each block; this was obtained by counting card picks from advantageous decks (C + D) minus the number from disadvantageous decks (A + B) in each block [i.e., (C + D) - (A + B)]. Higher results indicated better performance, while negative results indicated a preference for the disadvantageous decks.

##### RCFT

The RCFT is a test used to assess visual organisation, short-term visual memory and visuospatial abilities. The subject must copy and recall, after an interval of 3 minutes, a complex geometric figure. The accuracy of the reproduction of all the details of the figure was a measure of visuospatial and visual memory abilities. In addition, the RCFT can be used to examine organisational strategies used during the copy condition. It is possible to calculate a Central Coherence Index (CCI) that results from the order of construction index (drawing of global or local elements in the first stage of the copy task) and the Style Index (the degree of continuity in the drawing process). The CCI ranges from 0 (detailed) to 2 (global). Drawing style was assessed using both Savage’s [33] and Booth’s [34] scoring systems.

##### TMT

Cognitive flexibility was measured using a pencil-and-paper version of the TMT. The test was divided into two parts: in Part A, the subject must join with a continuous line the circles containing the numbers from 1 to 25 in ascending order; in Part B the subject must alternately join, always with a continuous line, a number (1 to 13) and a letter (A to N) that are found in circles in a random order on the page. The first part assessed visuospatial and motor skills of the subject; the second part assessed cognitive flexibility; more specifically, the time taken to carry out Part B of the task and the subtraction of time A from time B was considered a measure of cognitive flexibility.

##### WCST

The WCST was administered according to the Italian normative data [35]. It was used to assess the executive function deficits and required participants to match stimulus cards that vary in geometric shapes, colour and number of items per card. In the standard administration, the examiner does not inform the participant of the rule for correct matching (e.g., colour, shape, number of items on card) but simply responds ‘right’ or ‘wrong’ after each guess. In addition, the rule for correct matching changes without warning after the participant correctly matches 10 consecutive cards. The global score [number of trials – (number of achieved categories × 10)], Perseverative Errors, Non-Perseverative Errors, and Failures to maintain set were scored.

##### HSCT

The HSCT is a measure of response initiation and response suppression. We used the Italian adapted version of the HSCT [36] that consisted of 20 sentences in which the final word was missing; in Part A the examiner reads 10 sentences aloud and the participant has to simply complete each one, yielding a simple measure of response initiation speed. Part B requires subject to complete 10 sentences with a nonsense ending word (and suppress a sensible one), giving measures of response suppression ability and thinking time. Two practice sentences are read to the participants before each section. Participants are encouraged to respond as fast as they can. Other indexes provided by Part B are the type of answers: Type C Answer for sentence completion, Type S for semantic-related answers and Type U for semantic-unrelated answers. The sum of the Answers S and C provided the error score. The last index was the Average time of Type U answers.

All the neuropsychological measures were administered in the same session in this order: TMT, WCST, IGT, RCFT (copy), HSCT, RCFT (recall).

### Statistical analyses

Data were analysed using the Statistical Package for Social Sciences Version 21 (SPSS, Chicago, Illinois, USA). Anova followed by Bonferroni post hoc test was used to evaluate significant differences between AN, BED and HC. A univariate general linear model (UGLM) was used to check the influence of age, years of education, BMI and depressive symptoms (BDI score) on neuropsychological test performances. The first step was to evaluate differences in the neuropsychological tests for diagnosis and the second step was to evaluate the influence of age, education, BMI and BDI score as covariates. The level of statistical significance was set at p ≤ 0.05.

## Results

The sample consisted of 135 female participants, 45 per group.

### Clinical and demographic features of the sample

Table 1 describes the main characteristics of the sample, the scores of BDI and EDI-2 and the comparisons between groups. Significant differences were evident regarding age (F = 11.412; p < .001), years of education (F = 15.159; p < .001) and BMI (F = 289.725; p < .001) between groups and thus were included in the UGLM as covariates.

**Table 1:** **Demographic and clinical characteristics of the sample**

		**AN**	**BED**	**HC**	**Anova**		**Post hoc**
		**Mean** **±** **SD**	**Mean** **±** **SD**	**Mean** **±** **SD**	**F**	**Sig.**	
Age		22.8 ± 5.6	30.6 ± 10.9	25.6 ± 3.5	11.412	p < 0.001	BED > AN**; BED > HC*; AN < HC*
Education		12.2 ± 2.9	11.4 ± 2.6	14.8 ± 2.4	15.159	p < 0.001	BED = AN; BED < HC***; AN < HC**
BMI		15.5 ± 1.4	35.2 ± 6.5	20.2 ± 1.6	289.725	p < 0.001	BED > AN***; BED > HC***; AN < HC***
BDI		13.7 ± 6.8	27.8 ± 15.1	2.5 ± 3.3	70.543	p < 0.001	BED > AN***; BED > HC***; AN > HC***
EDI-2	DT	13.2 ± 6.7	13.0 ± 5.3	1.4 ± 3.3	64.010	p < 0.001	BED = AN; BED > HC***; AN > HC***
	B	2.3 ± 3.9	8.5 ± 6.6	0.9 ± 1.8	23.940	p < 0.01	BED > AN**; BED > HC***; AN = HC
	BD	13.1 ± 6.5	19.6 ± 5.3	4.3 ± 5.1	49.344	p < 0.001	BED > AN**; BED > HC***; AN > HC***
	I	10.2 ± 6.6	8.9 ± 5.5	1.8 ± 2.2	38.943	p < 0.001	BED = AN; BED > HC***; AN > HC***
	P	6.1 ± 3.5	3.6 ± 2.6	3.5 ± 3.7	5.975	p < 0.01	BED < AN*; BED = HC; AN > HC*
	ID	6.9 ± 4.3	4.9 ± 3.5	2.2 ± 2.5	17.248	p < 0.001	BED = AN; BED > HC*; AN > HC***
	IA	9.7 ± 6.1	9.2 ± 7.3	1.3 ± 3.3	33.007	p < 0.001	BED = AN; BED > HC***; AN > HC***
	MF	7.6 ± 5.8	8.2 ± 5.7	3.8 ± 3.7	7.059	p < 0.001	BED = AN; BED > HC**; AN > HC*
	ASC	7.6 ± 4.8	8.7 ± 3.7	2.2 ± 1.6	30.056	p < 0.001	BED = AN; BED > HC***; AN > HC***
	IR	6.7 ± 6.2	7.2 ± 6.1	1.3 ± 2.7	16.622	p < 0.001	BED = AN; BED > HC***; AN > HC***
	SI	7.8 ± 4.2	6.7 ± 3.3	2.4 ± 3.1	28.087	p < 0.001	BED = AN; BED > HC***; AN > HC***

*AN*: Anorexia Nervosa; *BED*: Binge Eating Disorder; *HC*: Healthy Controls; *BMI*: Body Mass Index; *BDI*: Beck Depression Inventory; *EDI*-2: Eating Disorder Inventory-2; *DT*: Drive for thinness; *B*: Bulimia; *BD*: Body dissatisfaction; *I*: Ineffectiveness; *P*: Perfectionism; *ID*: Interpersonal distrust; *IA*: Interoceptive awareness; *MF*: Maturity fears; *ASC*: Ascetism; *IR*: Impulse regulation; *SI*: Social insecurity.

*p < 0.05; **p < 0.01; ***p < 0.001.

Significant differences were also evident with regard to BDI (F = 70.543; p < .001): BED patients exhibited the highest scores in BDI corresponding to a moderate degree of depression, AN patients reported mild depression whereas HC reported minimum depression. Thus the BDI score was also included in the UGLM as covariate.

Regarding EDI-2, BED patients had significantly higher means in B and BD and lower P than AN patients and higher scores for all subscales of EDI-2 except for P compared to HC. Finally, AN patients had significantly higher means than HC in all EDI-2 subscales with the only exception of B.

Table 2 shows the results of the neuropsychological assessment and the comparison between groups.

**Table 2:** **Results of neuropsychological assessment**

		**AN**		**BED**		**HC**		**Anova**		**Post hoc**	**UGLM**		**Covariates** ^**a**^
		**Mean**	**SD**	**Mean**	**SD**	**Mean**	**SD**	**F**	**Sig.**		**F**	**p**	
IGT	Total score	−4.81	28.70	−8.40	32.92	11.92	31.32	5.028	<0.01	BED = AN; BED < HC*; AN < HC**	2.144	0.054	
	Block 1	−2.31	5.38	−1.80	6.58	−0.29	5.10	1.778	.173	BED = AN; BED = HC; AN = HC	1.026	0.412	
	Block 2	−2.31	8.03	−2.20	9.77	2.04	8.27	3.825	<0.05	BED = AN; BED = HC; AN < HC**	2.412	0.032	
	Block 3	−0.85	9.38	−1.90	9.10	3.84	9.62	4.146	<0.05	BED = AN; BED < HC*; AN < HC*	1.454	0.201	
	Block 4	0.62	9.05	1.00	9.50	3.33	8.47	1.262	.287	BED = AN; BED = HC; AN = HC	1.107	0.363	
	Block 5	0.04	9.05	−3.50	7.13	3.10	9.07	4.268	<0.05	BED = AN; BED < HC*; AN = HC	2.146	0.054	
HSCT	Part A	0.394	0.193	0.246	0.169	0.333	0.140	5.674	<0.01	BED < AN**; BED < HC*; AN = HC	1.439	0.206	
	Part B	1.878	1.328	2.348	1.086	1.514	1.072	3.632	<0.05	BED = AN; BED > HC**; AN = HC	2.042	0.066	
	Part B – Part A	1.508	1.304	2.099	1.017	1.175	1.034	4.589	<0.01	BED = AN; BED > HC***; AN = HC	2.229	0.046	Age
	Total errors	3.154	2.623	4.500	2.782	2.388	2.456	4.801	<0.01	BED = AN; BED < HC**; AN = HC	1.895	0.088	
	Type C answers	0.077	0.269	0.100	0.308	0.020	0.143	1.123	.329	BED = AN; BED = HC; AN = HC	0.839	0.542	
	Type S answers	3.077	2.480	4.400	2.703	2.327	2.401	4.994	<0.01	BED = AN; BED < HC**; AN = HC	2.278	0.041	
	Type U answers	6.846	2.531	5.550	2.704	7.653	2.411	5.067	<0.01	BED = AN; BED < HC**; AN = HC	2.190	0.049	
	Average time Type U answers	2.172	2.205	3.758	2.883	2.016	2.864	3.449	<0.05	BED > AN*; BED < HC*; AN = HC	2.568	0.023	Age, BDI
TMT	Part A (msec)	36.01	17.40	35.20	13.50	32.60	9.18	.786	.458	BED = AN; BED = HC; AN = HC	1.845	0.097	
	Part B (msec)	74.34	31.58	99.13	46.96	66.49	16.36	8.500	<0.001	BED > AN*; BED > HC***; AN = HC	3.891	0.001	Age, education
	Errors in Part B	0.38	0.69	0.50	1.05	0.14	0.35	2.735	.069	BED = AN; BED > HC*; AN > HC*	1.333	0.249	
	Part B – Part A (msec)	38.35	21.96	63.93	39.99	35.10	13.40	11.668	<0.001	BED > AN***; BED > HC***; AN = HC	5.109	<.001	Age, education
WCST	Global score	20.69	12.10	49.85	27.80	14.61	4.48	46.307	<0.001	BED > AN***; BED > HC***; AN > HC***	17.959	<.001	BMI, diagnosis
	Perseverative errors	5.58	2.48	5.50	2.63	4.73	1.54	2.089	.128	BED = AN; BED = HC; AN > HC*	2.692	0.018	
	Non perseverative errors	7.79	4.70	25.50	18.11	5.06	2.63	47.843	<0.001	BED > AN***; BED > HC***; AN > HC***	19.290	<.001	BMI, diagnosis
	Failures to maintain set	0.31	0.58	0.85	0.75	0.10	0.37	13.747	<0.001	BED > AN**; BED > HC***; AN > HC*	4.107	0.001	diagnosis
RCFT	Accuracy	35.79	0.61	34.95	2.09	35.88	0.33	7.274	.001	BED < AN**; BED < HC**; AN = HC	5.848	<.001	age, BMI, BDI, education, diagnosis
	Order	1.25	1.01	1.42	0.93	1.61	1.10	1.570	.212	BED = AN; BED = HC; AN = HC	1.539	0.172	
	Style	1.17	0.74	1.36	0.57	1.42	0.68	1.733	.181	BED = AN; BED = HC; AN = HC	1.799	0.106	
	Central Coherence Index	0.96	0.64	1.10	0.54	1.20	0.66	1.766	.176	BED = AN; BED = HC; AN = HC	1.754	0.116	
	Organization Strategies	2.94	2.25	3.90	2.15	3.84	2.51	2.250	.110	BED = AN; BED = HC; AN = HC	3.121	0.007	
	Percentage of recall	63.18	17.95	59.71	17.77	68.03	14.83	2.076	.130	BED = AN; BED < HC*; AN = HC	2.738	0.016	

*AN*: Anorexia Nervosa; *BED*: Binge Eating Disorder; *HC*: Healthy Controls; *IGT*: Iowa Gambling Task; *HCST*: Hayling Sentence Completion Test; *TMT*: Trial Making Task; *WCST*: Wisconsin Card Sorting Test; *RCFT*: Rey-Osterrieth Complex Figure Test; *UGML*: univariate general linear model.

^a^Covariates: only variables that result significantly associated are displayed.

*p < 0.05; **p < 0.01; ***p < 0.001.

### Decision making

**Figure 1:**
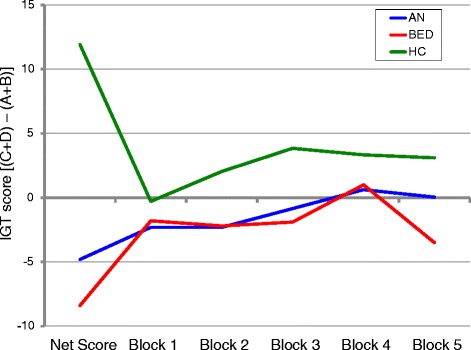
**Iowa Gambling Task:** **performance in Eating Disorder groups and Healthy Controls.** Mean net score and net number of chosen cards [(C + D) – (A + B)] across the five blocks each consisting of 20 trials by AN, BED and HC groups. Positive scores reflect advantageous performance whereas negative scores indicate the opposite.

- IGT. Total Score was significantly lower for BED and AN patients compared to HC participants. Furthermore, BED performed significantly lower than HC on Blocks 3 and 5 while AN performed significantly lower than HC in Blocks 2 and 3 (Figure 1).

### Cognitive flexibility

- HCST. BED performed better than AN and HC in Part A of HCST; contrarily BED had a significantly lower performance in Average time of Type U answers compared to both AN and HC. They also had poorer performance than HC in Part B, Part B-Part A and provided more type S and U answers. Interestingly no significant differences were found between AN and HC on HSCT. Differences between groups remained significant in Part B-Part A when correcting for age, and in Average time Type U answers when correcting for age and BDI scores.
- TMT. Significant differences emerged between AN and BED patients compared to HC in the number of errors in Part B. Moreover, BED patients performed worse than AN and HC in Part B and Part B-Part A. These last differences remained significant between groups when correcting for age and years of education.
- WCST. BED patients also had the worst performance in all indexes except on perseverative errors compared to AN and HC; AN performed significantly worse than HC in all subscales. Differences between groups remained significant in Global score, Non perseverative errors and Failure to maintain the set when correcting for diagnosis; when analysis were corrected for BMI, the differences in Global score and Non perseverative errors remained significant.

### Central coherence

- RCFT. BED had the lowest score on Rey-Accuracy Index compared to AN and HC in the copy condition of the RCFT; furthermore they had a significantly poorer performance on RCFT Percentage of recall than HC. The differences between groups remained significant after controlling for age, BMI, BDI score, years of education and diagnosis.

## Discussion

The aim of this study was to assess and compare BED and AN patients on the three most widely studied domains of cognition through neuropsychological testing. BED and AN patients showed high rates of cognitive impairment compared to HC on the domains investigated; furthermore the profile of BED patients was characterised by a poorer performance in decision making along the test, poorer cognitive flexibility and lower accuracy during the RCFT compared to patients with AN.

To our knowledge this is the first study to compare BED and AN patients on a wide range of neuropsychological tests (i.e., decision-making, set-shifting and central coherence).

Regarding decision-making strategies, our results were in line with recent literature on the performance of BED [12,14] and AN [3,37] patients. In fact, our data showed that BED had lower Total Score and lower values in the 3^rd^ and the 5^th^ Block (and thus worse results), indicating a difficulty in finding a correct strategy for the test. In agreement with other authors [12], BED patients often made more risky decisions than HC, showing impaired capacities to advantageously utilise feedback processing. Instead AN patients compared to HC group had lower Total Score and lower values in the second and third blocks of IGT, denoting greater insensitivity to feedback in early stages of the test.

HSCT was previously used only twice in the field of EDs [3,38] and BED patients were not involved. In our study, the most interesting results were those regarding BED who did Part A faster than AN and HC, yet they provided more Type S answers and took longer to give Type U answers in Part B. The results in Part A may be explained by their higher impulsivity, a typical trait associated with BED [39,40]; instead, the lower performance in Part B reflected more severe cognitive flexibility impairment. Furthermore, although no significant differences emerged between AN and HC, AN performed worse in all indexes. Significantly worse performance compared to HC in Type S and U answers have been described among AN patients [3].

Regarding TMT, BED patients were significantly slower than AN and HC in TMT Part B and proportional score Part B-Part A, according to another study [12], that indicated lower executive functioning among BED. Duchesne et al. [14] pointed out that BED obese hardly reached significant differences from non-BED obese; others found no differences on cognitive functioning performances between morbidly obese individuals with and without BED [15] so authors concluded that ‘obesity rather than binge eating, may be directly related to cognition’. In our study, AN patients had a worse performance in the number of errors on Part B compared to HC. Even if TMT is widely used in the literature, it provides controversial results [3,41-43] so another explanation may be given. Perfectionism, a characteristic psychological AN trait, leads to cognitive rigidity; nevertheless it can be of advantageous help for AN patients as it furthers the drive for good performance through a higher cognitive effort [44] which was not found among other ED patients.

Interesting results emerged in WCST. Research has demonstrated a worse performance by AN patients compared to HC [3,45,46]; our results were in accordance with those findings. In fact AN patients performed worse than HC group in all subscales. Nevertheless BED group was revealed to be the most impaired: they failed to find an appropriate strategy, as demonstrated by the high number of non-perseverative errors and the number of failures to maintain set. It could be read as if BED followed a trial-and-error strategy. On the other hand, AN patients committed more perseverative errors demonstrating more cognitive rigidity. Other authors [14] found not only a greater difficulty to maintain the set but also more perseverative errors in obese with BED than in obese controls; this last result should be better addressed comparing BED obese patients with AN and normal-weight HC in order to ascertain if this is a real or a relative increment of perseverative errors.

Finally, significant differences emerged on RCFT. Although this test was previously used with AN patients [41,47-50], this was the first time it was applied in the context of BED. BED patients showed a lower score on Rey-Accuracy index compared to AN and HC in the copy condition; furthermore BED had a significantly poorer performance on RCFT percentage of recall than HC. Even if our results were not statistically significant they showed a trend towards significance and thus they seem to be in line with previous studies [41,43,47,48] confirming that AN patients have lower central coherence (e.g., low order, style and central coherence) than HC. It was as if they drew their copy based on the details of the picture rather than oriented to it globally.

Thus, poor central coherence seems to be the characteristic feature of AN patients while poor attention, rather than lack of central coherence, is the distinctive trait of BED patients.

In this study depression interfered with cognitive performance as it negatively correlated with cognitive flexibility; in our case BED patients showed longer average time of Type U answers in HCST that could be explained by slowed thinking - a characteristic symptom of patients with depression. Cognitive impairments are common in Major Depression [51] and reflect the general inability to concentrate [8,52]. An inverse correlation between depression severity and cognitive performance in domains of executive function among others has been reported [53] and studies have demonstrated how depression also interferes with cognitive performance among ED patients [3,54-57]. The present results confirmed this influence in patients with BED. Possible explanations can be different. On the one hand, BED patients were more severely depressed, and depression is a frequently comorbid disorder in this ED [10,58-60]; on the other hand, BED participants were older and had less education. Age is related to lower cognitive flexibility and slower information processing that might explain a longer reaction time to TMT and HCST [61,62]. Education may reflect the skill level that patients acquired through the school years [63]. Further, people with depression are less attentive to details and the low RCFT Accuracy among BED patients could support it.

Body weight (BMI) was significantly associated with poor cognitive flexibility (WCST) in the sense that extreme weight conditions performed worse than HC. More specifically BED performed worse than AN, and AN worse than HC. Fagundo et al. [6] also found that AN and OB made significantly more errors than controls and had significantly fewer correct responses in WCST.

Although our findings provided a pattern of cognitive impairment for AN and BED, the present study was limited in several ways. The first limit could be the lack of an obese non-BED control group to better assess the cognitive profile across the extreme eating/weight conditions, and in greater detail between obese BED and non-BED [11-16]. Second, ours was a cross-sectional study, and results could change in the long term after therapy. Third, nutritional indexes (i.e., glycemia, hypertension, level of serotonin and dopamine) were not provided but there is evidence of their involvement in human cognition [64-66]. Finally, although contrasting data exist about the influence of anxious symptomatology on neurocognitive performance of ED patients [43,67-69], the present research did not take this variable into consideration; therefore future studies should include measures of anxiety symptoms to better clarify its effects, if any, on these domains.

## Conclusions

BED patients, and likewise other subjects with EDs, exhibited a pathological cognitive pattern. AN patients showed impaired cognitive flexibility, decision making and central coherence. Compared to patients with AN, the cognitive profile of BED was characterised by poorer decision making and cognitive flexibility. Thus, two different neurocognitive profiles emerged: a strong cognitive rigidity and a central coherence based on the details appeared predominant in patients with AN, while a lack of attention and difficulty in adapting to changes in a new situation seemed to better describe patients with BED. The knowledge of the different cognitive profiles of EDs patients may be important for the planning their psychotherapeutic intervention.

## Abbreviations

ED: Eating Disorder
AN: Anorexia Nervosa
BN: Bulimia Nervosa
EWC: Extreme weight condition
OB: Obese subjects
BED: Binge Eating Disorder
ADHD: Attention Deficit Hyperactivity Disorder
HC: Healthy controls
BMI: Body mass index
EDI-2: Eating Disorder Inventory 2
DT: Drive for thinness
B: Bulimia
BD: Body dissatisfaction
IA: Interoceptive awareness
ASC: Asceticism
P: Perfectionism
MF: Maturity fears
IR: Impulse regulation
IN: Ineffectiveness
SI: Social insecurity
ID: Interpersonal distrust
BES: Binge Eating Scale
BDI: Beck Depression Inventory
IGT: Iowa Gambling Task
HCST: Hayling Sentence Completion Test
TMT: Trial Making Task
WCST: Wisconsin Card Sorting Test
RCFT: Rey-Osterrieth Complex Figure Test
UGLM: Univariate General Linear Model
